# Editorial: Quantifying and Addressing Bias Associated With Imperfect Observation Processes in Epidemiological Studies

**DOI:** 10.3389/fvets.2019.00310

**Published:** 2019-09-13

**Authors:** Timothée Vergne, Julian A. Drewe

**Affiliations:** ^1^UMR ENVT-INRA 1225, Ecole Nationale Vétérinaire de Toulouse, Toulouse, France; ^2^Veterinary Epidemiology, Economics and Public Health Group, Royal Veterinary College, London, United Kingdom

**Keywords:** under-detection, bias, capture-recapture, misclassification, detection, imperfect, epidemiology, risk factors

If all data were accurate, the truth would be easier to find. However, epidemiological data used in most studies are generated by imperfect observation processes. For example, surveillance systems capture only a fraction of the true situation. Diagnostic tests that correctly identify all infected individuals are rare. Some subjects—such as wildlife and stigmatized groups of people—can be elusive and missed by researchers (1, 2). Finally, for social and economic reasons—such as when animals represent a family's livelihood—people can be reluctant to report disease outbreaks to the authorities (3). All these examples represent sources of bias which may have profound impacts on our understanding (and therefore management) of important diseases in animal and human populations.

Historically, these imperfect observation processes were often considered nuisances and ignored. In recent years, methodological developments such as the application of capture-recapture techniques (4), and the move toward interdisciplinary approaches, are beginning to allow us to properly account for these observation biases.

The aim of this Research Topic—which comprises eight papers spanning several disciplines—is to showcase latest developments in quantifying and addressing imperfect observation processes. We hope that the range of examples will encourage readers to look for the imperfections in their own observations, in order to identify—and if possible, control—biases when analyzing and interpreting data.

## Impact of Imperfect Observation Processes

Imperfect diagnostic tests may fail to detect some infected individuals and incorrectly classify some healthy ones as diseased. These are types of misclassification bias, the consequences of which can be severe. Combelles et al. examine the impact of imperfect disease detectability on the quantification of risk factors. They find that true group-level risk factors are generally correctly identified but that measures of association are heavily underestimated if the sensitivity of detection is not perfect. If the detectability of infected individuals also varies between groups, then variables associated with detection heterogeneity may erroneously be identified as risk factors.

In longitudinal studies, at-risk and incident cases can be wrongly identified, leading to selection and misclassification biases, respectively. Haine et al. use simulated data to investigate how these two types of bias may impact measures of incidence and risk ratio estimates in longitudinal studies. Their results suggest that modeling outcome biases are mainly related to test specificity and disease frequency, and emphasize that the tools used to identify at-risk and incident cases need to be carefully evaluated to make trustworthy inferences.

## Quantifying Imperfect Diagnostic Effectiveness

A versatile approach to quantifying the uncertainty around diagnostic test accuracy in the absence of a perfect reference test is to use Bayesian latent class inference. This is demonstrated by McDonald and Hodgson who explore the influence of both the precision and accuracy of prior estimates on the precision and accuracy of posterior estimates of diagnostic test performance and tuberculosis prevalence in a wild badger population in Britain. A key finding is that analyses can be conducted without a gold standard using imprecise priors, as long as models are initialized with accuracy.

Sometimes, diagnostic specificity needs to be high (for example, when a disease is rare or it is costly to deal with false positives). In such circumstances, the sample size required to estimate the true performance of a diagnostic test can become very large. This is shown by Rydevik et al. in their evaluation of tests with near-perfect specificity. Using simulations aiming at supporting the design of a diagnostic that is able to differentiate infected from vaccinated animals, the authors propose a two-stage approach to evaluating diagnostic tests in low disease prevalence populations.

## Accounting for Imperfect Observation Processes

Invasive wild animal species may be under-reported. In order to map high-risk areas for two aquatic invasive species in Minnesota, USA, Kanankege et al. used a co-kriging model to predict introduction locations without complete data; this helped identify risk factors for invasive species introduction. The authors claim that this method can be used as a solution to underreporting in ecological and epidemiological studies and improve early detection of biological invasions.

Understanding disease dynamics in wild animal populations represents a substantial challenge because these populations are often difficult to observe. In their paper, Lachish and Murray review all sources of potential uncertainty that can arise in studies estimating disease-related parameters in wild animal populations. This review should raise the awareness of researchers and practitioners regarding uncertainty in wildlife epidemiology studies.

To address different sources of uncertainty when studying disease dynamics in wildlife populations, capture-recapture models are known to be particularly relevant. However, it is unclear how much uncertainty these models can tolerate and still provide reliable estimates of population and disease dynamics. Using simulated data, Benhaiem et al. assess how estimates of survival probability, and the probability of transition between infection states, are affected by increasing uncertainty. They show that multi-event capture-recapture models are relatively robust to state uncertainty and heterogeneity in state assignment.

The important role of human behavior in the success—or otherwise—of disease surveillance and control programmes has only recently been realized and embraced in the field of veterinary epidemiology. Hidano et al. show that we need to update our thinking on this even further. Human behavior, including the inclination to report or seek treatment for disease, does not remain constant over time; it can change in response to an outbreak for which the behavior itself may have been modeled as part of disease control efforts. These authors conclude that, in order to solve these challenges, there is a need for interdisciplinary collaboration across a wide range of fields including animal health, animal welfare, epidemiology, and sociology.

## Conclusion

The eight papers showcase a range of approaches to quantifying and addressing bias associated with imperfect observation processes in epidemiological studies. Together, they confirm the ongoing need to acknowledge that imperfect observations and processes exist. We should not be afraid to investigate the potential impacts of such biases, and we should present and interpret the findings of such analyses in order to strengthen future research.

## Author Contributions

TV and JD defined together the Research Topic, edited the different papers and wrote the editorial.

### Conflict of Interest Statement

The authors declare that the research was conducted in the absence of any commercial or financial relationships that could be construed as a potential conflict of interest.
